# Effects of doxycycline on intrusive experimental trauma memory: a pre-registered, randomized double-blind placebo-controlled trial

**DOI:** 10.1038/s41398-025-03657-0

**Published:** 2026-03-09

**Authors:** Laura Meister, Alex Rosi-Andersen, Francesco Bavato, Yanfang Xia, Dominik R. Bach, Birgit Kleim

**Affiliations:** 1 Experimental Psychopathology and Psychotherapy, Department of Psychology, University of Zurich, Zurich, Switzerland; 2 Department of Adult Psychiatry and Psychotherapy, University Hospital of Psychiatry Zurich, University of Zurich, Zurich, Switzerland; 3 Institute of Toxicology and Pharmacology, University of Zürich, Zürich, Switzerland; 4 Donders Institute for Brain, Cognition and Behaviour, Radboud University Nijmegen, Nijmegen, the Netherlands; 5 Department of Psychiatry, Radboud University Medical Centre, Nijmegen, the Netherlands; 6 Neuroscience Center Zurich (ZNZ), University of Zurich, Zurich, Switzerland; 7 University of Bonn, Transdisciplinary Research Area Life & Health, Centre for Artificial Intelligence and Neuroscience, Bonn, Germany

**Keywords:** Human behaviour, Pathogenesis

## Abstract

A core clinical feature of posttraumatic stress disorder (PTSD) is recurrent reexperiencing of the traumatic event in the form of intrusive memories. Doxycycline is a matrix metalloproteinase 9 (MMP-9) inhibitor. MMP-9 is required for late-phase, NMDA receptor-dependent long-term potentiation in the hippocampus and the basal and central amygdala nuclei, which are important to various forms of learning and memory. Here we examined the effect of doxycycline on the development of intrusive memories in a pre-registered randomized, double-blind, placebo-controlled trial (https://osf.io/72ys9). Healthy females (N = 80) received 200 mg doxycycline or placebo 4.5 h before exposure to film footage depicting strong interpersonal violence. Participants then completed an intrusion diary for one week. Most participants, 92%, experienced intrusive memories following the trauma film. There was no evidence that doxycycline and placebo groups differed in frequency, distress, and vividness of daily intrusive memories models. The doxycycline group showed enhanced arousal, indexed by skin conductance when exposed to reminder cues, and better performance in a memory task about film content compared to placebo one-week post-film. Based on our findings, the MMP9-inhibitor doxycycline did not impair the development of intrusive memories and was associated with increased arousal and improved retrieval of experimental trauma memory one week later.

## Introduction

Recurrent, involuntary, and intrusive distressing memories of traumatic event(s) play a role in many psychological disorders and are a core feature of posttraumatic stress disorder (PTSD) [1]. Intrusive memories are common following trauma exposure, especially in the first few days and weeks, and are maintained in individuals who develop PTSD. Such intrusive memories have been identified as a central element in network models that are connected to other symptoms of the disorder (e.g., hyperarousal) and may thus easily activate them [2, 3]. Targeting intrusive memories after trauma has beneficial effects on other symptoms and may prevent the development of PTSD [4]. Intrusive memories are also the target of trauma-focused psychotherapy, a first-line treatment for PTSD [5].

In the last two decades, animal research significantly advanced our understanding of the development and malleability of memory as well as its modification, suggesting routes to secondary prevention of intrusive memories [6]. Memory consolidation is commonly conceptualized as a two-stage process. Its initial phase, synaptic consolidation, occurs within hours of learning and involves protein synthesis and structural modifications at the synapse. The subsequent phase, systems consolidation, unfolds over days to weeks and is characterized by gradual reorganization and redistribution of memory traces across brain networks [6, 7]. Synaptic consolidation has been intensively investigated through long-term potentiation (LTP), a widely used cellular model of memory [8, 9], and studies employing pharmacological interventions during this phase have demonstrated its critical role in long-term memory retention. [4, 10]. So far evidence for pharmacological agents in humans that interfere via blocking protein synthesis is limited. For example, propranolol, a β-adrenergic receptor antagonist, has been proposed to interfere with memory consolidation and reconsolidation by altering noradrenergic signaling rather than directly inhibiting protein synthesis [10–12]. Propranolol’s results on reducing fear memory and PTSD symptoms have been mixed and the exact neural underpinnings are unknown [13–15]. However, findings on the efficacy of propranolol on fear memory in humans are far from complete amnesia of fear memories as shown in animal studies using anisomycin [15]. While the direct inhibition of protein synthesis by compounds such as anisomycin produces desirable effects, they are not applicable in humans due to their intrinsic toxicity [14].

A promising alternative involves targeting matrix metalloproteinase-9 (MMP-9), a key enzyme in extracellular synaptic remodeling. MMP-9 is required for late-phase, NMDA receptor–dependent LTP in the hippocampus and the basal and central nuclei of the amygdala, regions critical for various forms of learning and memory [16–18]. Rather than inhibiting protein synthesis per se, MMP-9 inhibitors such as doxycycline appear to interfere with synaptic plasticity by preventing extracellular matrix degradation, a critical step in enabling structural synaptic changes [19, 20]. Doxycycline crosses the blood-brain barrier and has been shown to impair fear memory formation in humans when administered before fear conditioning [21]. However, its effects on more complex, autobiographical, and episodic intrusive memories, as triggered by real-life trauma or trauma analogs, remain unknown.

Here we investigate the effects of doxycycline on the development of intrusive memories following exposure to experimental trauma in the laboratory using a trauma film paradigm [22]. Specifically, we tested effects of doxycycline vs. placebo on the development of intrusive memories in a randomized, placebo-controlled, double-blind, trial. We hypothesized fewer intrusions and less intrusion-associated distress and vividness after experimental trauma in participants who received doxycycline compared to placebo (https://osf.io/72ys9). In addition to self-reported intrusions, we employed a multi-method approach to assess doxycycline effects on broader emotional and physiological responses associated with trauma memory formation. Traumatic memories engage distinct components- including emotional intensity, physiological arousal, and voluntary recall - supported by different neural systems. Prior trauma film paradigm studies have similarly incorporated such measures [22]. This approach aimed to capture the potential for pharmacological modulation to influence these separate facets of memory processing. Such findings would have implications for secondary prevention of intrusive memories after a traumatic event and could for example guide the treatment of patients after recent trauma in the emergency department.

## Materials and methods

### Participants

**Fig. 1:**
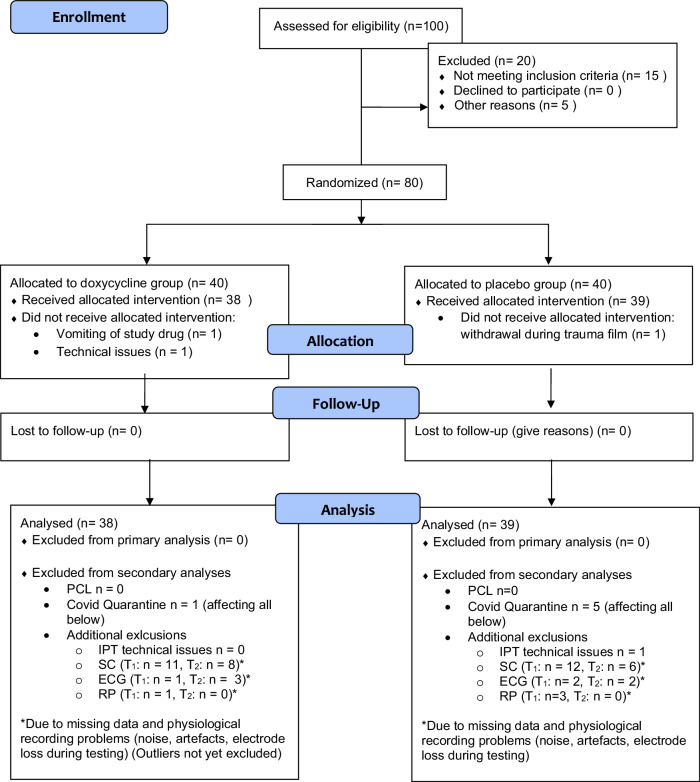
Study flow chart. PCL = PTSD Checklist for DSM-5. Intrusion Provocation Task = analyses of intrusions in the intrusion provocation task. T_1_ = Analyses of physiological data recorded at the experimental session (T_1_). T_2_ = analyses of physiological data recorded at follow-up (T_2_). SC = analyses of skin conductance. ECG = analyses of heart rate measures. RP = analyses of respiration period. (Values above Q3 + 1.5xIQR or below Q1 - 1.5xIQR were considered as outliers and excluded from the physiological data analyses which led to further exclusion in the analyses at T_2_ for RP (doxycycline: n = 3, placebo: n = 3), skin conductance variables SCL (doxycycline: n = 0, placebo: n = 2), AUC (doxycycline: n = 2, placebo: n = 3), number of spontaneous fluctuations by DCM (doxycycline: n = 4, placebo: n = 0), number of spontaneous fluctuations by MP (doxycycline: n = 5, placebo: n = 4) and ECG variables BPM (doxycycline: n = 3, placebo: n = 1), RMSSD (doxycycline: n = 7, placebo: n = 3), HF (doxycycline: n = 2, placebo: n = 0) and at T3 for RP (doxycycline: n = 3, placebo: n = 2), SCL (doxycycline: n = 2, placebo: n = 2), AUC (doxycycline: n = 3, placebo: n = 3), DCM (doxycycline: n = 8, placebo: n = 6), MP (doxycycline: n = 11, placebo: n = 8) and BPM (doxycycline: n = 2, placebo: n = 0), RMSSD (doxycycline: n = 2, placebo: n = 0), HF (doxycycline: n = 2, placebo: n = 1).

Participants were 80 healthy young females between 18 and 40 recruited from the general population via websites, flyers, and mailing lists. Inclusion criteria were physiological and psychological health, female sex. Participation was restricted to females due to the chosen analog trauma model. The film footage depicting interpersonal violence with a male perpetrator and a female survivor is likely to cause different effects on males and females that would be difficult to control in a mixed sample. Exclusion criteria were pregnancy, breastfeeding, any medication intake (except oral contraceptive and NSAR), regular consumption of media depicting sexual violence, and a lifetime history of interpersonal violence. All participants were screened for physical and psychological health conditions by a physician. Two participants did not complete the study: one due to vomiting after drug ingestion and one discontinued participation during the trauma film (see Fig. 1).

The study was conducted in accord with the Declaration of Helsinki and approved by the cantonal research ethics committee (Kantonale Ethikkomission Zurich, KEK-ZH 2018-01973) and the Swiss Agency for Therapeutic Products (Swissmedic, Bern, Switzerland; 2019DR1026). All participants gave written informed consent using a form approved by the ethics committee. Participants were compensated with approximately CHF 50 per hour which is the usual rate of reimbursement in Switzerland for pharmacological studies. Total study participation took around 8 h and was compensated with 400 CHF.

### Power analysis

To determine the required sample size, we conducted a power analysis based on previous studies with a similar setup with different pharmacological interventions (hydrocortisone and propranolol) [23]. Under the assumption of equal variance in a placebo- and a doxycycline-treated group and no variance due to the intervention, an intrusion reduction of ~50% would correspond to an effect size of (Cohen’s) d = 0.77. Thus, a sample size of N = 44 is required to achieve 80% power at an alpha rate of 0.05. To allow for the unknown heterogeneity of the drug effect and some attrition, we recruited N = 80 participants.

### Study medication

The tetracycline antibiotic agent doxycycline was used in this study, which is sold under the trade name Vibramycin (Arzneimittel-Kompendium, 2019). It is commonly prescribed to treat various infectious diseases, including Lyme disease and prophylaxis for malaria (Arzneimittel-Kompendium, 2019). The study dose of 200 mg is the smallest dose recommended by the manufacturer to minimize side effects [24], such as photosensitivity, nausea, and headache (Arzneimittel-Kompendium, 2019). After oral ingestion, we allowed for a metabolization period of 210 min before neuropsychological testing of memory and 270 min before film viewing, in order for the medication to reach peak level concentration in the CSF [25, 26]. The drug was manufactured, blinded, and randomized by the Kantonsapotheke, Zurich, Switzerland. Mannitol was used as a placebo.

### Procedure

**Fig. 2:**
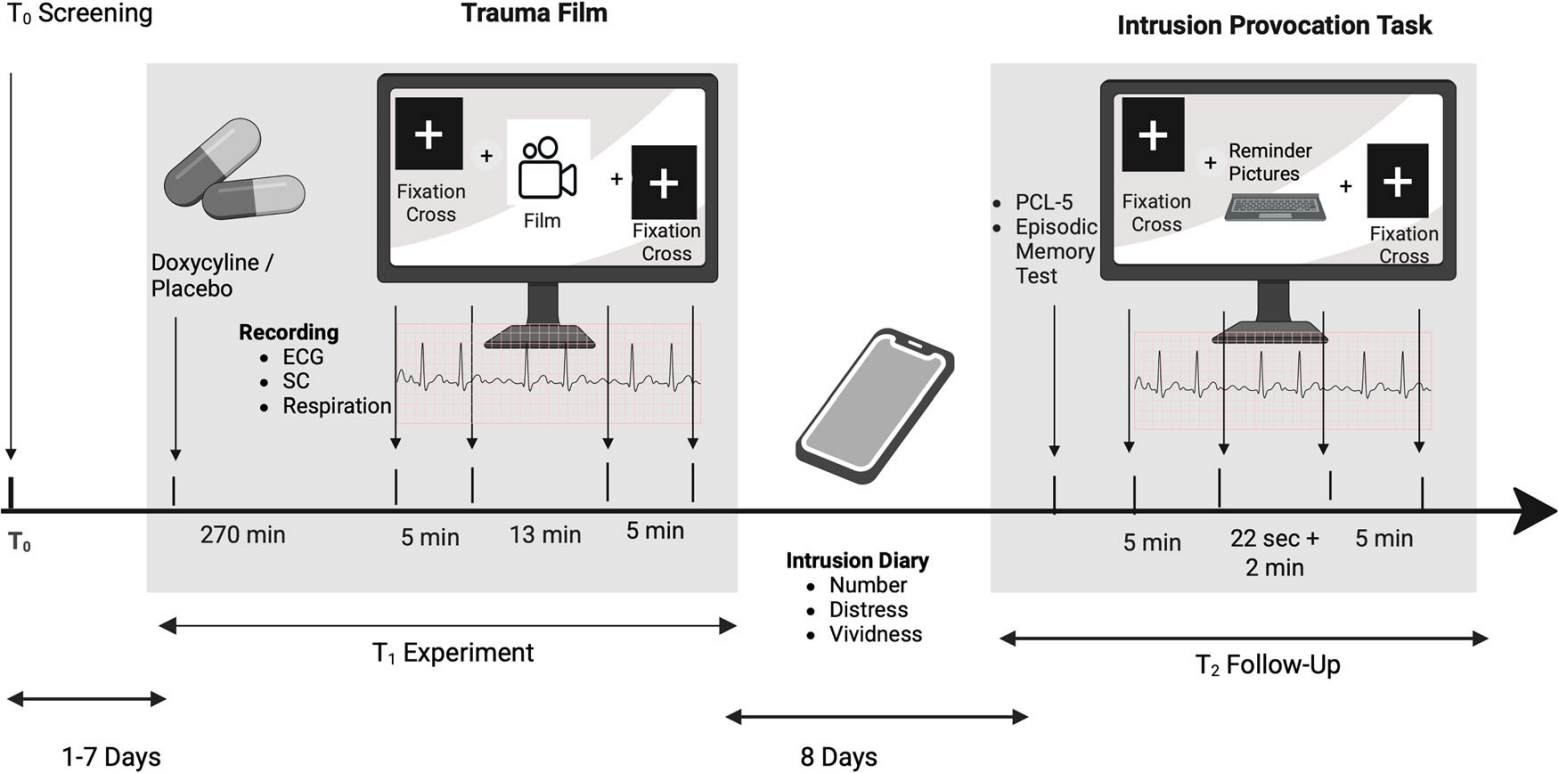
Study procedure. This figure depicts the experimental procedure. ECG = Electrocardiogram. SC = skin conductance. PCL-5 = Posttraumatic Check List. The intrusion provocation task consisted of 11 reminder pictures each shown for 22 s followed by a 2-min-period where participants had to close their eyes and indicate occurring intrusions by pressing a key on the keyboard. Figure in BioRender https://BioRender.com/iilfxiv.

The study consisted of three study visits (screening, experiment, and follow-up), see Fig. 2.

#### Screening (T_0_)

Participants gave written informed consent, were screened for inclusion and exclusion criteria, and filled in the Life Events Check List of the DSM-5 (LEC) and the Childhood Trauma Questionnaire (CTQ) [24] to verify that participants did not have a lifetime history of personal violence, the Beck Depression Inventory (BDI-II) [27], and the Beck Anxiety Inventory (BAI) [28] (see Table 1).

**Table 1:** Baseline sample characteristics (N = 78).

Variable		*M* (*SD*)		α	*U*	*z*	*p*
		Doxycycline	Placebo				
Age		23.050 (3.170)	23.590 (4.228)		780.5	0.201	0.841
BDI-II		2.510 (2.501)	1.870 (1.936)	0.64	680.5	–0.891	0.413
BAI		2.950 (2.946)	2.900 (3.050)	0.75	737.5	–0.234	0.815
CTQ		31.1 (6.28)	29.15 (3.59)	0.8	886	-1.255	0.209
Emotional Abuse		7.18 (2.53)	6.13 (1.52)	0.8	990	-2.377	0.017
Physical Abuse		5.03 (0.16)	5.05 (0.22)	na	741	-0.57	0.567
Emotional Neglect		8.08 (3.64)	7.08 (2.26)	0.84	850	-0.911	0.362
Physical Neglect		5.82 (1.6)	5.9 (1.43)	0.25	707	-0.615	0.539
Sexual Abuse		5 (0)	5(0)	na	na	na	na
STAI Trait		34.230 (6.466)	34.310 (7.034)	0.86	750	–0.105	0.916
I-PANAS-SF Trait							
Positive Affect		18.380 (2.642)	18.330 (2.216)	0.66	732	–0.287	0.774
Negative Affect		7.280 (2.460)	7.490 (2.151)	0.76	854	0.95	0.342
RTS	58.490 (13.843)		55.210 (15.487)	0.9	639.5	–1.210	0.226
PTCI		6.133 (2.616)	6.870 (2.704)	0.94	872	1.114	0.265
SWE		28.790 (3.450)	29.970 (3.468)	0.81	837	0.769	0.442
CERQ							
Functional		69.180 (9.624)	68.080 (9.059)	0.79	665	–0.955	0.339
Dysfunctional		36.180 (6.637)	36.210 (6.392)	0.75	702	–0.586	0.558
MEQ		50.21 (7.42)	50.29 (9.54)	0.84	737.5	-0.223	0.822
PSQI		1.85 (2.19)	2.03 (1.84)	0.74	847.5	-0.888	0.375
BFI-K							
Extraversion		3.417 (0.887)	3.333 (0.812)	0.83	716	–0.446	0.655
Agreeableness		3.314 (0.601)	3.372 (0.819)	0.64	795.5	0.352	0.724
Conscientiousness		3.756 (0.708)	3.910 (0.540)	0.7	780.5	0.202	0.84
Neuroticism		2.551 (0.669)	2.506 (0.689)	0.69	716.5	–0.443	0.658
Openness		3.954 (0.633)	4.077 (0.682)	0.75	861.5	1.014	0.31

α = Cronbachs α. U = Mann-Whitney-U-Test with standardized z-values and two-sided, asymptotic significance. Doxycycline n = 39, Placebo n = 39.

*BDI-II* revised beck depression inventory [27] (scale 0–63), *BAI* beck anxiety inventory [28] (scale 0–63), *STAI* state-trait anxiety inventory [29] (scale 4–80). *I-PANAS-SF* international positive and negative affect schedule short form [37] (scale 5–25). *RTS*, ruminative thought style questionnaire [62] (scale 15–105). *PTCI*, posttraumatic cognitions inventory [63] (scale 3–21). *SWE* skala zur allgemeinen selbstwirksamkeitserwartung [64] (scale 10–40). *CERQ*, cognitive emotion regulation questionnaire [30], functional = acceptance + positive refocusing + refocus + positive reappraisal + putting into perspective (scale 20–100), dysfunctional = self-blame + rumination + catastrophizing + other-blame (scale 16–80). *BFI-K* brief version of the big five inventory [65] (1-5 per scale). *CTQ*, childhood trauma questionnaire [24] consist of the five subscales emotional abuse, emotional neglect, physical abuse, physical neglect and sexual abuse (scale 5–25); *MEQ*, *Morningness-Eveningness Questionnaire* (Greifahn et al., 2001) (scale 14–86). *BIS-BAS*, behavioral approach and behavioral inhibition systems [66]. BAS consists of the three scales fun seeking, drive and reward responsiveness. *LEC*, life events checklist [67] *PSQI*, pittsburgh sleep quality index [68] (scale 0–21).

#### Experimental laboratory session (T_1_)

Participants were invited to a lab session between 7.00 am and 9.00 am to control circadian influences on pharmacological effects and film viewing. At the beginning of the experimental session, participants were randomly assigned to ingest 200 mg doxycycline or placebo. They filled in questionnaires including the Spielberger State-Trait Anxiety Inventory (STAI) [29] and the Cognitive Emotion Regulation Questionnaire (CERQ) [30]. After ingesting doxycycline vs placebo there was a subsequent wait of 210 min. Participants stayed at the psychiatric university hospitals and could choose what they wanted to do in the 210-min waiting period (for example relax, chat, eat little snacks). They were checked on every 30 min. They were not allowed to engage in sports, eat heavy meals or smoke as this could have interfered with drug absorption. We then conducted a series of neuropsychological tests to assess verbal and visual declarative memory and procedural memory independent of the film content [31–33], which we reported elsewhere [34]. Participants were then exposed to stressful film footage depicting strong interpersonal violence. The trauma film included scenes of interpersonal violence, selected based on prior evidence indicating its effectiveness in reliably inducing intrusive memories. We acknowledge the ethical imperative to minimize participant distress and selected this film with caution, following ethical approval and established protocols for monitoring and managing participant wellbeing. The decision reflects a trade-off between experimental reliability and ethical responsibility and underscores the need for continued discussion around appropriate analog stimuli in trauma research. The film consisted of a 13-minute scene from the movie “Irréversible” (directed by Gaspar Noé, 2002) frequently used in trauma analog studies [13, 35, 36]. Participants were instructed to sit still and watch the scene as if they were present as a witness. This took part in a darkened, quiet room. Participants placed their heads on a chin rest at 70 cm from the monitor (Dell P2012H, 20 inches, adjusted to an aspect ratio of 5:4, 60 Hz refresh rate).

We assessed respiration, heart rate, skin conductance, and employed eye-tracking during the film viewing. Before the trauma film, we assessed physiological data in a neutral rest condition. For this, participants were asked to relax and fixate on a white cross on a black screen for 5 min. We then assessed physiological arousal during the trauma film. Immediately after the trauma film, participants were again asked to relax and fixate on a white cross for 5 min.

Immediately before and after the film viewing participants filled out several questionnaires to control various peritraumatic experiences including the change in positive and negative affect assessed with the state version of the Positive and Negative Affect Schedule (PANAS-S) [37] and indicated their arousal on a subjective units of distress scale (1 = calm, 9 = excited).

#### Intrusive memory assessment in daily life: Ecological momentary assessment (EMA)

Following an initial explanation about intrusive memories and detailed instructions about keeping the intrusion diary, participants recorded every intrusive memory relating to the trauma film in an app-based intrusion diary. This data was collected through the smartphone app “SEMA3” (Smartphone Ecological Momentary Assessment) (https://sema3.com). The following variables were surveyed through the SEMA3-App for each intrusion: (a) content, (b) perceived distress on a scale of 0–10, (c) perceived vividness on a scale of 0–10, (d) type of memory (image, thought, or both), (e) suddenness, and (f) additional remarks. All entries were timestamped. The EMA structure was event-based. Participants filled in the intrusion diary whenever an intrusive memory occurred. Participants were asked to rate the accuracy of their intrusion diary on a scale of 1 to 100 as a measure of compliance [35].

#### Follow-Up (T_2_)

Seven days after the experimental visit, i.e., after drug wash-out, participants returned for a follow-up assessment. Participants completed an episodic memory test in which they answered questions about the trauma film. Participants then rated their intrusive reexperiencing in the last week by filling in the PTSD checklist from DSM-5 (PCL-5). We also repeated the neuropsychological tests to assess delayed recall and recognition in verbal, visual, and procedural memory.

An intrusion provocation task was performed [38]. Stimuli for the intrusion provocation task consisted of 11 blurred static visual images created using a Gaussian blur filter (Adobe Photoshop CS6, version 13.06 ×64). The images were taken from scenes in the first 2 min of the movie before the traumatic events started [39]. The images were presented for 2 s each in chronological order (picture viewing). Participants were then asked to close their eyes and think of nothing for 2 min. They were asked to press the space bar on the keyboard every time an intrusive memory of the trauma film popped up in their mind (intrusion monitoring) [40]. The total frequency of intrusive memories yielded an intrusion provocation task intrusion score. To assess physiological arousal pre-, during, and post- intrusion provocation task we obtained the same indices (heart rate, skin conductance, and respiration) as during film viewing. The time window during which the intrusion provocation task was kept between 8.00 am and 11.00 am.

The appointment ended with a short debriefing, and the participants were compensated with 400 CHF or credit, respectively.

The randomization code was broken after the last participant completed the study, and after all the data were checked for consistency.

### Psychophysiological assessment

Physiological data were assessed with Coulbourn Instruments digitized using a DI-149 A/D card (Dataq Instruments, Akron) and recorded with Windaq software (Dataq Instruments) [25, 41]. A detailed description can be found on https://osf.io/72ys9. Changes in heart rate were assessed with changes in beats per minute (BPM) and with heart rate variability (HRV). We assessed HRV with the root mean square of successive differences between normal heartbeats (RMSSD) and high-frequency power of frequency activity in the 0.15 – 0.40 Hz range (HF), both of which reflect vagal tone [42]. Changes in skin conductance were measured using skin conductance level (SCL), area under the curve (AUC), and number of spontaneous fluctuations (SF) calculated with the matched pursuit (SF-MP) and dynamic causal modeling (SF-DCM) algorithm [43]. Respiration period (RP) was used to analyze changes in respiration [41]. Analyses were pre-registered. We did not control for multiple comparisons across the different physiological measures.

Viewing direction was recorded with the EyeLink 1000 software (https://www.sr-research.com/eyelink-1000-plus/).

Film viewing and intrusion provocation task were programmed using the Cogent toolbox (version 2000v1.32, www.vislab.ucl.ac.uk/Cogent) in MATLAB (version R2019b, Math Works). To preprocess the data, we used PsPM (Psychophysiological modeling, http://pspm.sourceforge.net, version 5.1.1), a MATLAB toolbox for model-based analysis of psychophysiological data.

### Episodic memory Test

To assess participants’ memory about the content of the trauma film, we conducted an episodic memory test adapted from James et al. (2015) [38]. It comprised a set of seven open-format and eight true or false questions about the trauma film. Two raters evaluated the open-format questions. Each correctly answered question was awarded one point. An additional point was awarded for a more detailed answer to one of the open-format questions (“What was the protagonist wearing?”). A total score of 16 could be obtained. Inter-rater reliability was high, with kappa = 0.86.

### Data analysis

The analysis plan was pre-registered on the Open Science Framework osf.io/72ys9. We used RStudio (Version 4.0.3) for all analyses. Significance level for all analyses was set to α = 0.05.

To index film effects on subjective arousal and emotions, we calculated a Wilcoxon test comparing these indices pre- vs post-film across all participants. To investigate physiological fear responses to the trauma film and to account for group differences at baseline, we conducted a linear mixed effects model (group = doxycycline, placebo; time = pre-, during, post-film).

To test doxycycline’s effects on diary-based intrusion frequency, distress, and vividness, we conducted linear mixed effects models. Group x time analyses were calculated for intrusion frequency with the glmer() function and for vividness and distress with the lmer() function of the “lme4” package (group = doxycycline, placebo, time = days 1-8 post- film). We used Poisson regression with the log link function for intrusion frequency due to the right-skewed count data [44]. To test effects of doxycycline on questionnaire-based intrusive reexperiencing (PCL-5), we used multiple linear regression models using lm() and quasipoisson regression to test effects on intrusion frequency in the intrusion provocation task [44]. Covariates were selected based on previous research indicating that depressive symptoms, trait anxiety, and affective state are associated with post-traumatic responding and the development of intrusive memories [4, 45].

We departed from our pre-registered study plan to better account for the distributional properties of our data and used linear mixed effects models, Poisson and Quasipoisson regressions instead of ANCOVA. We chose a Poisson regression for intrusion frequency due to the right-skewed count data [44]. Using Poisson regression, no log-transformation was required, and the probability of alpha and beta errors could be minimized. We used mixed-effects model to account for clustering and handling missing data more appropriately. Model diagnostics supported the use of a Poisson model over a negative binomial alternative (lower AIC, no overdispersion, minimal zero inflation; see Table S1 in the supplementary materials). To test the effects of doxycycline on intrusion count in the intrusion provocation task, the Quasipoisson regression was preferred to the standard Poisson regression to counteract overdispersion of the data [44]. In the intrusion diary, there was no overdispersion of the intrusion count data. Vividness and distress scores were calculated as the average daily ratings per participant during the intrusion monitoring period.

To test effects of doxycycline on physiological response (heart rate, skin conductance, respiration) during intrusion provocation, we used linear mixed-effects models using lmer() (Group = doxycycline, placebo, time = pre-, during, and post-intrusion provocation task (picture viewing and intrusion monitoring)). The analysis of physiological measures was based on average values calculated for each time point. The reported effects reflect time-specific levels rather than change scores from baseline. Outliers in physiological response indices were identified with the first (Q1) and third quartile (Q3) and the interquartile range (IQR = Q3-Q1). Values above Q3 + 1.5xIQR or below Q1-1.5xIQR were considered outliers and excluded from the physiological data analyses. Post-hoc, we conducted t-tests to test for group differences (doxycycline vs placebo) in physiological response indices in each intrusion provocation task condition (pre-, during picture viewing, during intrusion monitoring, and post). Significant results were Holm-Bonferroni corrected (see supplementary materials).

## Results

### Participant characteristics and blinding

The sample comprised 78 female participants with a mean age of 23 years (SD = 3.7), of which 39 received placebo and 39 doxycycline. The groups did not differ in age or any other demographic and clinical variables, except for more self-reported emotional abuse in doxycycline compared to placebo, see Table 1. Participants were unaware if they were in the placebo or doxycycline group (χ2 = 0.510, p = 0.475). Some side effects were reported (nausea, doxycycline: n = 3), tachycardia (doxycycline: n = 1), vertigo (doxycycline: n = 1), stomach pain (doxycycline: n = 1), headache (doxycycline: n = 1), and fatigue (placebo: n = 1)). In the doxycycline group, 7 out of 38 participants (18.4%) reported side effects, compared to 1 out of 39 participants (2.6%) in the placebo group. A Chi-square test of independence indicated no statistical significance, χ²(1, N = 77) = 3.63, p = 0.057.

### Subjective and physiological response to experimental trauma

**Fig. 3:**
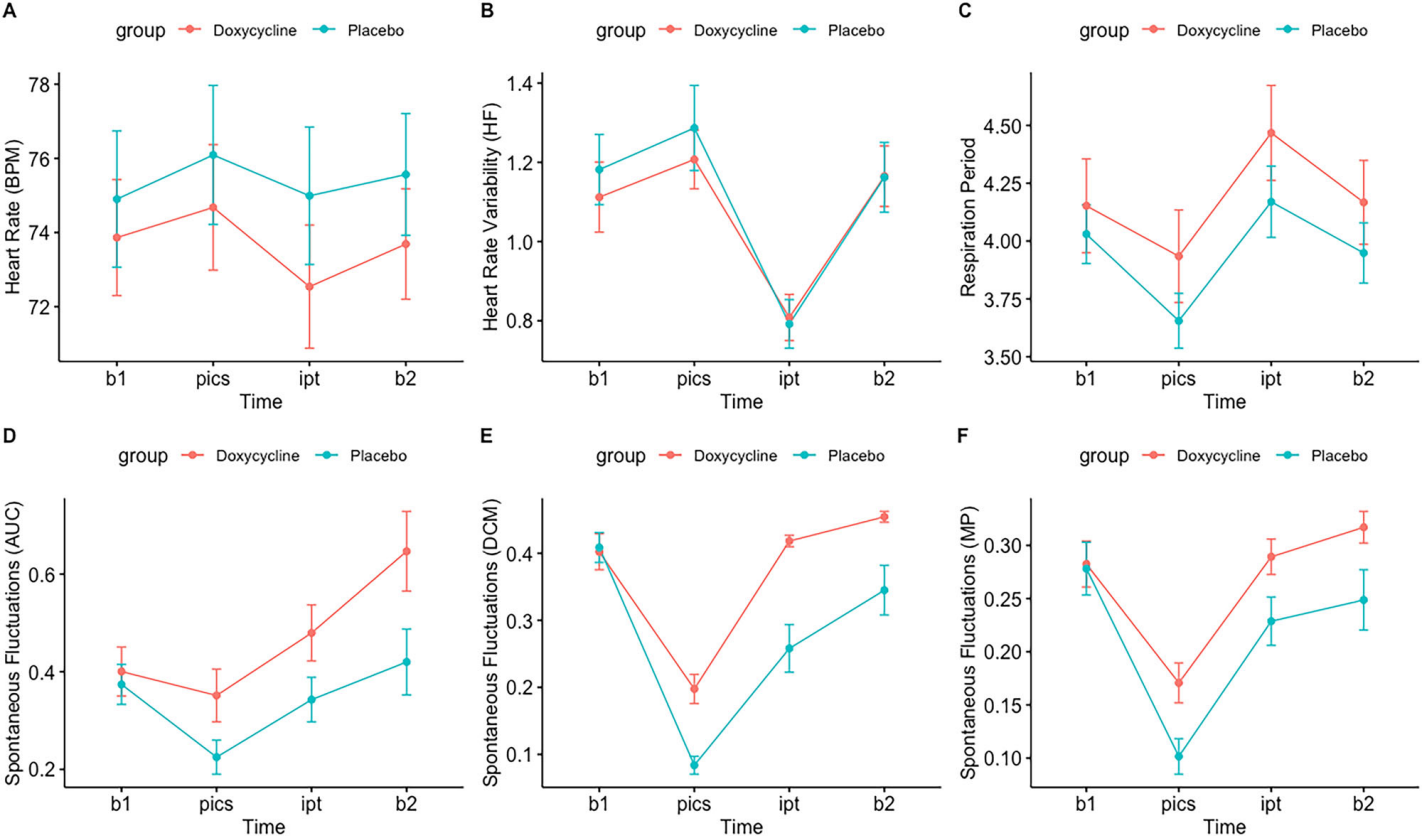
Physiological arousal during trauma film paradigm. Heart rate (**A**), heart rate variability (**B**), respiration period (**C**), spontaneous fluctuations (AUC) (**D**), spontaneous fluctuations (DCM) (**E**), spontaneous fluctuations (MP) (**F**) assessed during the trauma film paradigm. AUC = skin conductance area under the curve (microsiemens). BPM = heart rate beats per minute. DCM = number of spontaneous fluctuations of skin conductance per second calculated with the dynamic causal modelling algorithm. HF = heart rate variability (high frequency). MP = number of spontaneous fluctuations of skin conductance per second calculated with the matched pursuit algorithm. RMSSD = heart rate variability (root mean square of successive differences between normal heartbeats). RP = respiration period, duration of a breathing cycle in seconds. SCL = skin conductance level (microsiemens). b1 = 5-min fixation cross immediately before task. film = 13-min trauma film. b2 = 5-min fixation cross immediately after task. Red line = doxycycline. Blue Line = placebo. Vertical lines = standard errors.

Participants reported more negative emotions post- compared to pre- film (PANAS: V = 3, z = −7.55, p < 0.001). Subjective arousal increased significantly post- compared to pre- film (SUDS: V = 17, z = −7.40, p < 0.001). Participants also showed a heightened physiological response, observed in shorter respiration period, higher skin conductance, higher heart rate and lower heart rate variability during film compared to rest (RP: F(1, 2) = 53.10, p < 0.001), SCL: F(1, 2) = 19.41, p < 0.001), AUC (F(1, 2) = 24.18, p < 0.001), SF-DCM (F(1, 2) = 21.09, p < 0.001), SF-MP (F(1, 2) = 4.54, p = 0.0134), BPM(F(1, 2) = 6.49, p = 0.002), HF: (F(1, 2) = 89.96, p < 0.001)) (Fig. 3). For RMSSD no significant time effect was found.

### Intrusions

Participants rated their compliance in diary keeping high on average, 83% (SD = 15.48). Exclusion of participants with compliance lower than 80% (n = 17) did not influence reported results. The overall sample reported a total of 267 intrusions in the diary, of which 206 were visual. Most participants, 90.91%, reported at least one intrusive memory. On average, participants experienced 3.47 (SD = 3.59 [R = 0 – 21]) intrusive memories over the eight days after the trauma film. Participants reported a mean distress of 2.99 (SD = 1.72) afflicted with their intrusions. Average vividness was 3.57 (SD = 2). Number, distress, and vividness decreased over the course of the week, with all p-values < 0.001, see Table 2.

**Table 2:** Multilevel model investigating time x group interactions in predicting diary-based intrusion characteristics (N = 78).

Intrusion:	Frequency			Distress			Vividness		
*Predictors*	*Incidence Rate Ratios*	*CI*	*p*	*Estimates*	*CI*	*p*	*Estimates*	*CI*	*p*
(Intercept)	0.86	0.23 – 3.20	0.824	3.49	1.06 – 5.91	0.005	4.01	1.14 – 6.89	0.007
time	0.71	0.65 – 0.78	0.001	−0.25	−0.44 – -0.07	0.007	−0.25	−0.47 – -0.02	0.031
group [Placebo]	1.11	0.62 – 1.96	0.731	−0.86	−2.02 – 0.31	0.147	−0.59	−1.99 – 0.82	0.411
Depression (BDI)	1.00	0.89 – 1.13	0.959	−0.12	−0.33 – 0.08	0.242	−0.04	−0.29 – 0.20	0.739
Trait Anxiety (STAI)	1.00	0.96 – 1.04	0.820	0.03	−0.04 – 0.10	0.406	0.02	−0.07 – 0.11	0.649
Positive Affect (PANAS-S)	0.98	0.92 – 1.04	0.442	−0.03	−0.14 – 0.07	0.570	−0.06	−0.18 – 0.07	0.380
Negative Affect (PANAS-S)	1.06	1.00 – 1.11	0.044	0.10	−0.00 – 0.20	0.052	0.09	−0.03 – 0.21	0.123
time * group [Placebo]	1.01	0.89 – 1.15	0.836	0.15	−0.10 – 0.40	0.248	0.09	−0.21 – 0.40	0.546
**Random Effects**									
σ^2^	1.38			1.72			1.63		
τ_00_	0.48 _ID_			2.43 _ID_			4.55 _ID_		
τ_11_				0.05 _ID.time_			0.13 _ID.time_		
ρ_01_				−0.58 _ID_			−0.67 _ID_		
ICC	0.26			0.52			0.66		
N	74 _ID_			67 _ID_			67 _ID_		
Observations	592			181			181		
Marginal R^2^ / Conditional R^2^	0.255 / 0.446			0.122 / 0.576			0.082 / 0.684		

Table contains results of the three mixed effects models used to test effects of doxycycline on daily intrusion frequency (Poisson regression with glmer (glmer(freq_intrusions ~ time + group + time:group + (1 | ID) + BDI + STAI + Positive Affect + Negative Affect, family = “poisson”, data=long, control=glmerControl(optimizer = “bobyqa”, optCtrl=list(maxfun=100000))))) and on associated distress and vividness (linear mixed effects model with lmer (lmer(vividness resp. distress ~ time*group + (1+time|ID) + BDI + STAI + Positve Affect + Negative Affect, data=long))). Depression, trait anxiety, and negative and positive mood changes from pre- to post film viewing were considered as predictors.Time = 8 days of the intrusion diary. Group = doxycycline or placebo. Group [Placebo] is referring to the reference group. Incidence rate ratio (IRR) > 1 indicate a positive, IRR ≤ 1, a negative association, i.e., Time = IRR of 0.71 is indicating a decrease of daily intrusion frequency over time.

R^2^= percentage of variation in the response explained by the model. Marginal R^2^ = proportion of variance explained by the fixed factors alone. R^2^ conditional = proportion of variance explained by both the fixed and random factors. Sigma^2^ = within-group (residual) variance. Tau_00_ = between-group-variance (variation between individual intercepts and average intercept). Tau_11_= random-slope-variance (variation between individual slopes and average slope). Rho_01_= random-intercept-slope-correlation. ICC = intraclass-correlation coefficient, proportion of the variance explained by the grouping structure in the population.

*BDI* becks depression inventory [27], *STAI* state-trait anxiety inventory [69], *PANAS-S* [37] positive and negative affect scale state version, *CI* confidence interval.

In the IPT, participants reported 3.86 intrusions on average (SD = 3.04) with most participants, 91.43%, indicating at least one intrusive memory during the task (n = 64). Mean subjective distress was moderate, 2.11 (SD = 1.92) as was the vividness of these intrusions 2.84 (SD = 2.22).

### Effects of Doxycycline on intrusion, episodic memory, and psychophysiological outcomes

**Fig. 4:**
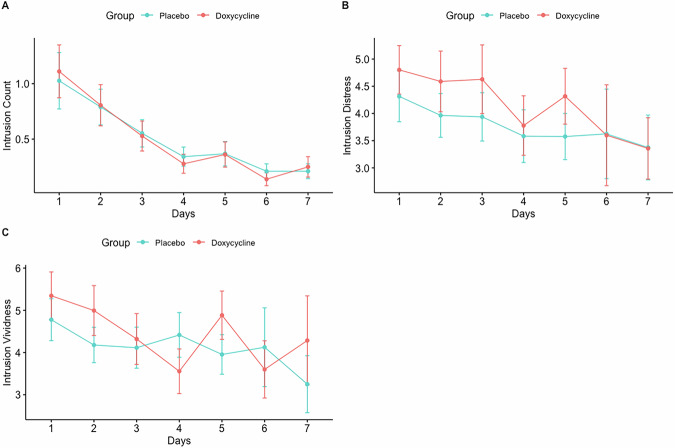
Intrusion count, vividness and distress in the diary. Daily intrusion count (**A**), distress (**B**) and vividness (**C**) of intrusions entered in the intrusion diary. Vertical lines are standard errors. The red line depicts the doxycycline group. The blue line depicts the placebo group.

Our pre-registered primary analyses revealed no significant difference between doxycycline and placebo in frequency and slope of daily intrusive memories, see Table 2 and Fig 4. Time and increase of negative affect during the film, were both significant predictors for intrusion frequency, see Table 2, indicating that a higher increase of negative emotions during the film led to more intrusive memories and that these decreased over time.

Doxycycline and placebo groups also did not differ in vividness, distress, or slope of daily vividness and distress in a linear mixed effects model, see Table 2 and Fig. 2. To account for baseline group differences, we included childhood emotional abuse (CTQ) as a covariate in the linear mixed effects models, which did not lead to changes in results, except for negative affect which was then a significant predictor for daily distress (b = 0.11, CI[0.00 – 0.21], p = 0.041), but not for daily intrusion frequency (IRR = 1.05, CI[1.00 – 1.11], p = 0.064).

There was no significant group difference in reexperiencing assessed with the PCL-5 (see Table 3).

**Table 3:** Regression of the reexperiencing subscale of the PCL-5 (N = 76).

Predictor	*b*	*b* 95% CI [LL, UL]	*sr*^*2*^	*sr*^*2*^ 95% CI [LL, UL]	Fit
(Intercept)	0.48	[−1.81, 2.77]			
Group [Placebo]	0.17	[−0.65, 1.00]	0.00	[−0.02, 0.02]	
Depression (BDI)	0.01	[−0.21, 0.23]	0.00	[−0.00, 0.00]	
Trait Anxiety (STAI)	0.00	[−0.07, 0.07]	0.00	[-0.00, 0.00]	
Positive Affect (PANAS-S)	−0.12*	[−0.24, -0.01]	0.06	[−0.05, 0.17]	
Negative Affect (PANAS-S)	0.13*	[0.03, 0.22]	0.10	[−0.03, 0.23]	
					*R*^*2*^ = 0.134
					95% CI[0.00,0.24]

A significant *b*-weight indicates the semi-partial correlation is also significant. *b* represents unstandardized regression weights. *sr*^*2*^ represents the semi-partial correlation squared. *LL* and *UL* indicate the lower and upper limits of a confidence interval, respectively.

*BDI* becks depression inventory [27], *STAI* state-trait anxiety inventory [69], *PANAS-S* [37] positive and negative affect scale state version, *CI* confidence interval

*indicates *p* < 0.05.

Participants showed good episodic memory of the film content overall, with 81% correct responses (SD = 9.37%) and better performance in the doxycycline group compared to the placebo group (U = 930.5, *p* = 0.046, r = 0.019).

There was no significant difference in intrusion frequency between the doxycycline group and the placebo group in the IPT, see Table 4, nor in associated distress and vividness (all p-values > 0.05).

**Table 4:** Quasipoisson regression of the intrusion score of the Intrusion Provocation Task (N = 70).

	IPT		
*Predictors*	*Incidence Rate Ratio*	*CI*	*p*
(Intercept)	1.62	0.61 – 4.28	0.332
Group [Placebo]	1.12	0.742 – 1.39	0.404
Depression (BDI)	1.07	0.98 – 1.18	0.137
Trait Anxiety (STAI)	1.01	0.98 – 1.03	0.723
Positive Affect (PANAS-S)	0.96	0.91 – 1.01	0.091
Negative Affect (PANAS-S)	1.08	1.04 – 1.12	0.000
Observations	70		
R^2^ Nagelkerke	0.435		

Incidence rate ratios (IRR) > 1 indicate a positive, IRR ≤ 1, a negative association. I.e., IRR of 1.08 in negative affects indicates a higher increase of negative emotions from pre- to post film viewing is associated with a higher number of intrusions in the intrusion provocation task. R^2^ Nagelkerke = approximated percentage of variation in the response explained by the model.

*BDI* becks depression inventory [27], *STAI* state-trait anxiety inventory [69], *PANAS-S* [37] positive and negative affect scale state version, *CI* confidence interval.

**Fig. 5:**
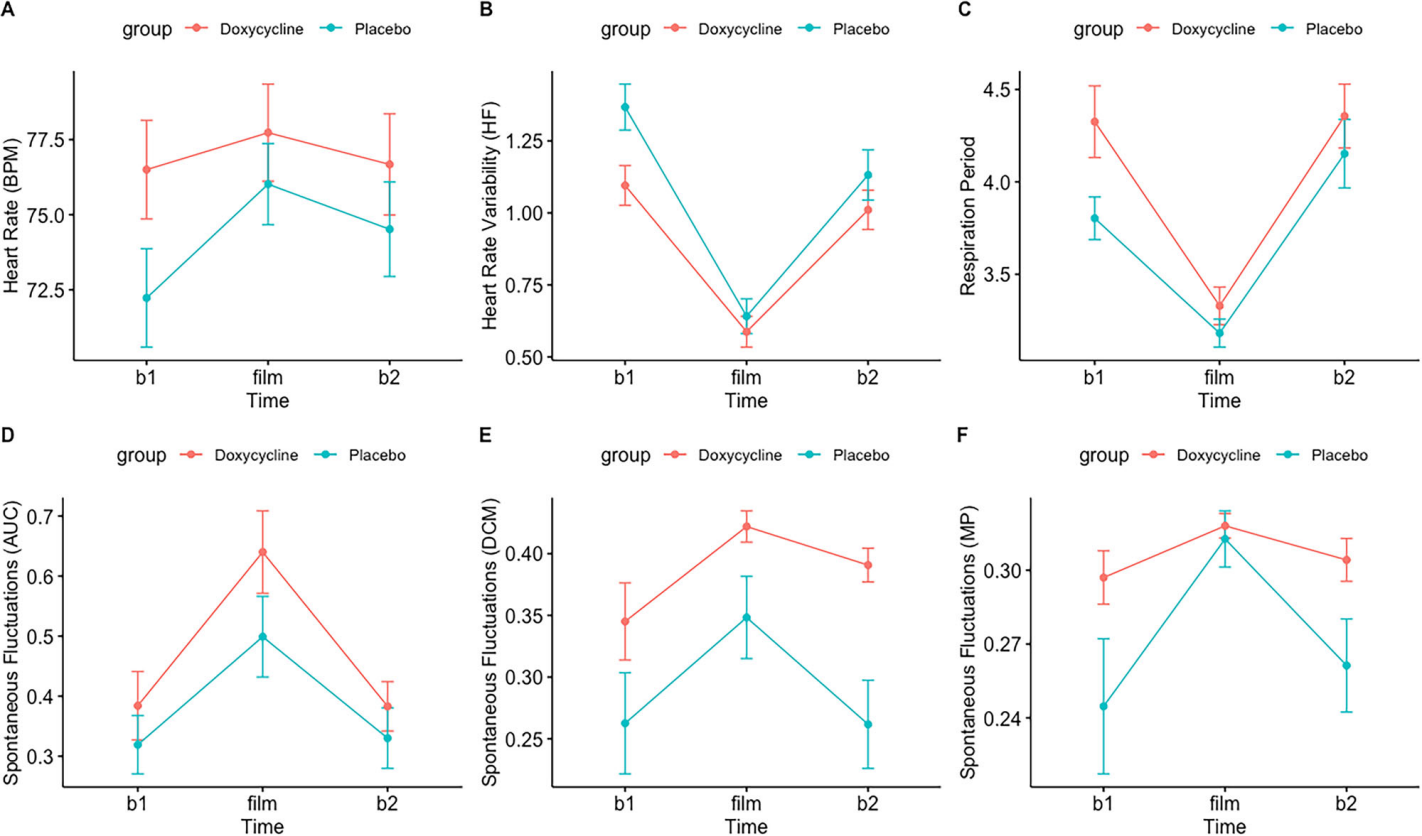
Physiological arousal during intrusion provocation task. Heart rate (**A**), heart rate variability (**B**), respiration period (**C**), spontaneous fluctuations (AUC) (**D**), spontaneous fluctuations (DCM) (**E**), spontaneous fluctuations (MP) (**F**) assessed during the intrusion provocation task. AUC = skin conductance area under the curve (microsiemens). BPM = heart rate beats per minute. DCM = number of spontaneous fluctuations of skin conductance calculated with the dynamic causal modelling algorithm. HF = heart rate variability (high frequency). MP = number of spontaneous fluctuations of skin conductance per second calculated with the matched pursuit algorithm. RMSSD = heart rate variability (root mean square of successive differences between normal heartbeats). RP = respiration period, duration of a breathing cycle in seconds. SCL = skin conductance level (microsiemens). b1 = 5-min fixation cross immediately before task. pics = 22-sec of reminder images in chronological order. intrusion provocation task = 2-min-phase of intrusion monitoring with eyes closed. b2 = 5-min fixation cross immediately after task. Red line = doxycycline. Blue line = placebo. Vertical lines = standard error.

In terms of psychophysiology, there was a main effect of group for AUC (F(1,3) = 4.24, p = 0.045), and a significant group per time interaction for SF-DCM (F(1,3) = 16.82, p < 0.001) and SF-MP F(1,3) = 4.02, p = 0.009) (Table S2 and S3 in the supplementary materials). The doxycycline group showed a higher AUC (t(44) = 2.298, p = 0.026) and higher number of spontaneous fluctuations (SF-DCM (t(35) = 2.682, p = 0.011, and SF-MP (t(33,9) = 3.484, p = 0.001) when exposed to trauma film reminders compared to the placebo group (Fig. 5). Doxycycline and placebo groups did not differ in in heart rate, heart rate variability, skin conductance level, nor respiration period during the intrusion provocation task (p > 0.05).

## Discussion

Our study investigated effects of doxycycline on experimental trauma memory consolidation and the development of intrusive memories. In our preregistered analyses, doxycycline and placebo groups did not significantly differ in intrusion frequency, vividness, distress or slope of these memories, indicating that doxycycline did not have the hypothesized effect on experimental trauma memory consolidation. There was also no group difference in self-reported reexperiencing symptoms at one-week post-experimental trauma. Participants in the doxycycline group did not show signs of smaller physiological stress reaction one week after the trauma film when exposed to reminder cues compared to the placebo group. On the contrary, participants in the doxycycline group had a higher number of spontaneous fluctuations of skin conductance when exposed to reminder cues, indicating increased response. Compared to the placebo group, the doxycycline group scored significantly higher in an episodic memory test assessing memory for details of the trauma film, indicating improved episodic memory consolidation.

Previous work has identified the matrix metalloproteinase-inhibiting drug doxycycline as a possible inhibitor of human fear memory consolidation [24]. This work tested effects of doxycycline on cued delay fear conditioning. Cued delay fear conditioning is assumed to mainly involve the amygdala, but not wider neural networks including the hippocampus [46] which plays a central role in the development of intrusive memories probed through the trauma film paradigm and real-life trauma [47, 48]. The purpose of our study was to investigate effects of doxycycline on episodic experimental trauma memories and their spontaneous reactions as well as conscious retrieval. Here we probed intrusive memories which are more hippocampus-dependent and involved contextual learning. The fact that hippocampus-based memories were mostly unaffected by doxycycline is in accord with recent studies [49], which did not replicate effects of doxycycline on trace fear conditioning and suggested that effects in healthy individuals are smaller and less robust than anticipated. Doxycycline attenuates memories that are amygdala-dependent but might not attenuate memories that include wider neural networks including the hippocampus [25]. Doxycycline is a MMP-9 inhibitor but may unfold various effects [50], for instance on other MMPs [17], hence influencing other relevant processes. Dosage was kept equal across participants and may have not been sufficient to exert large enough effects on key outcomes in some participants. Additionally, doxycycline was administrated at a single time point to target consolidation. Future studies may consider increasing the dosage or administration frequency to improve effects and target other memory processes such as reconsolidation [51]. Lastly, floor effects might have contributed to the lack of difference between groups in intrusion frequency. It is possible that pre-film memory tasks increased cognitive load and may have indirectly influenced the frequency of intrusions. While working memory interference effects are typically observed when tasks follow trauma exposure, we cannot rule out the possibility that our task timing contributed to the relatively low intrusion rates observed [22, 39, 52]. Intrusion frequency, vividness, and distress were, however, comparable to other trauma film paradigm studies [23, 35, 39, 53]. In the intrusion provocation task at one week, we did not identify distinct physiological fear responses to the trauma reminders. While we identified faster breathing, i.e., shorter RPs, during exposure to trauma reminders and lower heart rate variability, heart rate did not change across conditions and number of spontaneous skin conductance fluctuations decreased during exposure to trauma reminders. As the intrusion provocation task phase contained 11 reminder pictures, each presented for 2 s, the picture-evoked SCR might suppress SF [54]. While we aligned our intrusion provocation task with a previous study [38] to ascertain comparability across research labs, future studies could use stronger trauma reminder cues., i.e., showing the beginning of the scene to elicit a greater response.

We observed a significant group difference between the placebo and doxycycline groups in the performance in the episodic memory test, with better performance under doxycycline. This is interesting as strengthening accurate conscious memory of the details surrounding experimental trauma might be beneficial for secondary prevention of intrusive memories [55]. Ideal memory interventions may attenuate intrusive memories and physiological reactions while leaving voluntary episodic memory intact [4]. Although there is no previous data on effects of doxycycline on episodic memory, our findings align with a previous study observing an association between lower MMP-9 expression and higher performance in a declarative memory task [56]. However, doxycycline seemed to have simultaneously strengthened episodic memory and physiological responses captured in responding to trauma reminders later. MMP-9 is thus not specific to acting on memory formation in the amygdala but is also present in other brain regions and the peripheric nervous system and may have widespread effects on body and brain. Such effects are not yet fully understood in the context of memory, and, in the present study, there were significant effects relating to experimental trauma memories, specifically regarding episodic retrieval, whereas effects on involuntary intrusive memories remained nonsignificant. Some memory effects may be difficult to capture in the context of more complex trauma memories, and existing effects may thus be disguised. Although side effects were more frequently reported in the doxycycline group, they were generally mild and did not appear to have resulted in increased subjective distress or anxiety. Thus, while the impact of side effects on memory performance cannot be ruled out entirely, we consider it unlikely that side effects alone account for the observed differences in explicit memory.

Limitations of our study must be considered in interpreting the results. First, our study did not include a clinical population, and we cannot make assumptions about the efficacy of doxycycline for symptoms after real-life traumatic experiences. Second, our sample comprised only female participants. Emotional memory consolidation differs between men and women and female sex hormones impact the formation of intrusions [57, 58]. Future studies should include both sexes and control for sex hormones [59]. Third, the intrusion dairy relies on self-report and participants may underestimate intrusion frequency despite our clear instructions about intrusions and participants’ tracking of their intrusions on their smartphones. Additionally, while we assessed accuracy of the intrusion diaries, we did not assess it for the lab-based intrusions. Fourth, although vividness and distress ratings were averaged per participant to account for variability in intrusion frequency, we acknowledge that this approach may not fully capture the nuances of individual differences in intrusion experiences. While more complex multilevel models could provide finer resolution, we opted for a parsimonious linear mixed-effects approach to maintain model stability and interpretability given our sample size. Fifth, given the exploratory nature of several analyses, particularly in the physiological and explicit memory domains, we conducted multiple statistical tests without formal correction for multiple comparisons. This increases the risk of Type I error, and therefore, these findings should be interpreted with caution. Future studies should aim to replicate these results in larger samples and consider applying appropriate adjustments for multiple testing. Finally, we chose to administer doxycycline orally 4.5 h before the trauma film. Duration of action and the time of administration of doxycycline did thus not allow for discrimination between the effects of doxycycline during encoding or consolidation of the trauma film on the formation of intrusive memories. Similar studies using propranolol pointed out the added value of study designs comparing pre-learning and post-learning drug application [15]. To account for the pharmacokinetics of doxycycline we chose to administer it before the trauma film to have reached peak level during the trauma film [26].

In conclusion, experimental trauma exposure under doxycycline was not associated with attenuated frequency, distress, or vividness of intrusive memories compared to placebo. Doxycycline was associated with higher skin conductance and better performance in an episodic memory test one week after experimental trauma, indicating enhanced memory in the doxycycline group. These results implicate that doxycycline may affect memory consolidation, but, against our hypothesis, may improve certain memory features, but not others. The overall pattern of results does not confirm the hypothesis that doxycycline before trauma exposure attenuates trauma memory consolidation and intrusion formation. While our findings do not support the use of doxycycline for reducing intrusive memories in this context, the observed effects on explicit memory suggest that pharmacological interventions may selectively influence distinct components of trauma memory. Importantly, these results underscore the need for more systematic, hypothesis-driven studies to clarify the mechanisms and potential therapeutic value of such approaches. At this stage, our findings should be seen as a step toward refining, rather than endorsing, pharmacological adjuncts to trauma-focused interventions. Our research adds to the discussion of MMP-9 inhibitors as a potential pharmacological option to treat trauma-related disorders. More research is needed to fully comprehend the relationship between MMP-9 and aversive memory. Future research should identify and exploit pharmacological targets with suitable pharmacokinetics, specifically those that are more potent than doxycycline in inhibiting MMP-9 and with higher specificity for MMP-9 v other MMPs [60]. For example, minocycline is also an MMP-9 inhibiting drug and a tetracyclic antibiotic that is equipped with a more suitable pharmacokinetic; it crosses the blood-brain barrier faster than doxycycline and is more specific to MMP-9 [61]. Successful pharmacological approaches to trauma memory modulation could help secondary prevention of PTSD after a traumatic event and provide evidence-based guidance for the treatment of acutely traumatized patients in the emergency department.

## Supplementary information

Supplementary Material

## Data Availability

Anonymized data are available from the corresponding author upon reasonable request.
