# Supplementary material for: Effects of doxycycline on intrusive experimental trauma memory: a pre-registered, randomized double-blind placebo-controlled trial
Source: Transl Psychiatry. 2026 Mar 9;16:172. doi: 10.1038/s41398-025-03657-0 (PMC13039345; doi:10.1038/s41398-025-03657-0)
Supplement: Supplementary file 1 — Supplementary Material [file 41398_2025_3657_MOESM1_ESM.docx]

**Effects of doxycycline on intrusive experimental trauma memory:
A pre-registered, randomized double-blind placebo-controlled trial**

Laura Meister^1,2^, Alex Rosi-Andersen^1,2^, Francesco Bavato^2^, Yanfang Xia^2,3^, Dominik R. Bach^2,3,4^, Birgit Kleim^1,2,3^

^1^Experimental Psychopathology and Psychotherapy, Department of Psychology, University of Zurich, Zurich, Switzerland

^2^Department of Psychiatry, Psychotherapy and Psychosomatics, Psychiatric University Hospital Zurich, Zurich, Switzerland

^3^Neuroscience Center Zurich (ZNZ), University of Zurich, Zurich, Switzerland

^4^Hertz Chair for Artificial Intelligence and Neuroscience, University of Bonn, Germany

**Supplementary Material**

## MATERIALS AND METHODS

*Pre-Registration*

A detailed description can be found on https://osf.io/72ys9.

## DATA ANALYSIS

We used Poisson regression for intrusion frequency due to the right-skewed count data with log as the link function (1). Using Poisson regression, no log-transformation was required, and the probability of alpha and beta errors could be minimized. In case of non-convergence of the mixed effects models with the default optimizer, we used the bobyqa optimizer and chose the first converging model. In case no model converged, the random effects structure was not considered further.

For post-hoc comparisons, we used the emmeans package and employed Holm-Bonferroni correction. We corrected for 15 comparisons when assessing physiological data during the trauma film paradigm and for 28 comparisons during the intrusion provocation task. Only corrected significance levels are reported. We did not account for multiple dependent variables in our investigation of physiological fear memory.

*Used R Codes for Mixed Effects Models*

#install.packages

library("lme4")

library("Matrix")

library(qcc)

library(rstatix) # identify outliers

library(afex) # mixed-effects models

library(emmeans); library(multcomp) # post-hoc tests

library(effsize) # cohen's d

library(sjPlot) # make nice model table

#R-Code for Diary-Based Analyses

## Mixed Effects Model

fit1<- glmer(freq_intrusions ~ time + group + time:group + ( 1 + time | ID ), family = "poisson", data=long)

summary(fit1)

#Co-variates: Most common predictors (depression, anxiety, changes in pos. and changes in neg.affect during analog trauma)

fit2 <- glmer(freq_intrusions ~ time + group + time:group + ( 1 |ID ) + BDI_sum + STAI_sum + P.Affect_Diff1 + N.Affect_Diff1, family = "poisson", data=long, control=glmerControl(optimizer="bobyqa", optCtrl=list(maxfun=100000)))

summary(fit2)

#R-Code for Physiological Data

##Control for Outliers

ctrlOutlier <- scl2 %>%

group_by(group, Time) %>%

identify_outliers(scl)

##Mixed Effects Model

lmer_Physio <- lmer(formula = physio ~ Time * group + (1 | ID),

data = physiological_data)

anova(lmer_physio)

summary(lmer_physio)

posthoc_physio <- emmeans(lmer_physio, c("Time", "group"))

update(pairs(posthoc_physio), by = NULL, adjust = "holm")

summary(posthoc_physio)

*Physiological Data Analyses*

We used skin conductance responses (SCR) as a well-established index of sympathetic arousal and emotional reactivity, particularly relevant in PTSD research (1). In addition to conventional measures (SCL, AUC), we included model-based metrics using SF-MP and SF-DCM to better capture phasic arousal. SF-DCM in particular provides robust estimates of evoked responses in tasks with short inter-stimulus intervals and accounts for the long-tailed nature of SCRs (2). This physiologically informed approach improves sensitivity and interpretability of stimulus-related autonomic responses.

The high frequency (HF) power values reported in our manuscript are normalized values, expressed as percentages (%). HF power is divided by the total power (up to 0.4 Hz) excluding the VLF component and multiplied by 100: pHF=HF/(TP−VLFHF) ×100.

This normalization ensures that the reported values reflect the proportion of autonomic activity in the high-frequency band, independent of inter-individual variability in total heart rate variability (HRV) power. To check if linear modeling is appropriate, we assessed the normality of residuals from our mixed-effects model using a Q-Q plot (see figures A and B in the appendix). The residuals closely followed the theoretical normal distribution, with only minimal deviations at the extremes, and did not indicate a violation of model assumptions. Given that linear mixed-effects models are known to be robust to such minor departures from normality (3,4), our approach seems appropriate.

## RESULTS

*Model Comparisons for Primary Outcome Count Data*

**Table S1**. Model Comparison Between Poisson and Negative Binomial Mixed Models

| **Model** | **Distribution** | **AIC** | **BIC** | **Log Likelihood** | **Dispersion Ratio** | **Zero Counts (n, %)** |
| --- | --- | --- | --- | --- | --- | --- |
| fit2 | Poisson | **908.81** | **948.26** | -445.41 | 0.74 | 9 (1.5%) |
| fit2_nb | Negative Binomial | 910.81 | 954.65 | -445.41 | — | 9 (1.5%) |
| **Note**. The Poisson model (fit2) was fit using the glmer() function from the **lme4** package, and the Negative Binomial model (fit2_nb) using the glmmTMB() function. The dispersion ratio was derived from a Pearson chi-square test on the Poisson model, indicating slight underdispersion (<1). Zero inflation modeling was not pursued due to the low frequency of zero counts in the data (1.5%). Although the log-likelihood values are identical, the Poisson model demonstrated a marginally better fit in terms of AIC and BIC. | | | | | | |

*Physiological Change During Intrusion Provocation Task*

There was no increase in negative emotions and self-reported distress from pre- to post IPT, all p > 0.05). Participants showed a heightened physiological response to exposure to reminder pictures of the trauma film in the IPT observed in a lower RP, higher SCL and lower HF compared to the rest condition before and a general increase of AUC over the task (see table 1 and 2). No main effects of time were observed for BPM and RMSSD and the number of spontaneous fluctuations skin conductance as assessed via SF-DCM and SF-MP decreased from the rest condition to exposure to reminder pictures, see Table 1.

| **Table S2. Mixed effects models of physiological response to the intrusion provocation task (IPT)** | | | | | |
| --- | --- | --- | --- | --- | --- |
| ***Variable*** | ***Main Effect*** | ***DenDF*** | ***NumDF*** | ***F value*** | ***P(>F)*** |
| AUC | Time | 147 | 3 | 12.91 | 0.000 |
|  | Group | 49 | 1 | 4.22 | 0.045 |
|  | Group x Time | 147 | 3 | 2.14 | 0.098 |
|  |  |  |  |  |  |
| BPM | Time | 192 | 3 | 1.83 | 0.142 |
|  | Group | 64 | 1 | 0.07 | 0.787 |
|  | Group x Time | 192 | 3 | 0.99 | 0.399 |
|  |  |  |  |  |  |
| SF-DCM | Time | 123 | 3 | 69.95 | 0.000 |
|  | Group | 41 | 1 | 16.82 | 0.000 |
|  | Group x Time | 123 | 3 | 5.72 | 0.001 |
|  |  |  |  |  |  |
| HF | Time | 180 | 3 | 25.16 | 0.000 |
|  | Group | 60 | 1 | 0.79 | 0.377 |
|  | Group x Time | 180 | 3 | 0.26 | 0.855 |
|  |  |  |  |  |  |
| SF-MP | Time | 136 | 3 | 41.05 | 0.000 |
|  | Group | 46 | 1 | 4.95 | 0.031 |
|  | Group x Time | 136 | 3 | 2.03 | 0.112 |
|  |  |  |  |  |  |
| RMSSD | Time | 183 | 3 | 4.05 | 0.008 |
|  | Group | 61 | 1 | 0.18 | 0.674 |
|  | Group x Time | 183 | 3 | 0.20 | 0.899 |
|  |  |  |  |  |  |
| RP | Time | 174 | 3 | 11.85 | 0.000 |
|  | Group | 58 | 1 | 1.13 | 0.292 |
|  | Group x Time | 174 | 3 | 0.40 | 0.751 |
|  |  |  |  |  |  |
| SCL | Time | 156 | 3 | 6.49 | 0.000 |
|  | Group | 52 | 1 | 0.00 | 1.000 |
|  | Group x Time | 156 | 3 | 2.57 | 0.057 |
| *Note.* Table contains main effects of mixed effects model with lme4 (lmer(data ~ group*time + (1\|ID)). Time = Effects over different conditions of the INTRUSION PROVOCATION TASK (rest condition, trauma film reminders, intrusion monitoring, rest condition). Group = doxycycline vs. placebo. Group x Time = interaction: Interaction between treatment group and task phase, indicating whether the pattern of physiological change across time differs between groups. A significant interaction (p < .05) suggests that the two groups respond differently over time to the IPT, whereas a non-significant interaction suggests similar temporal response patterns across groups.DenDF= degrees of freedom in the denominator (sum of squares within). NumDF= degrees of freedom in the numerator (sum of squares between).  AUC = skin conductance area under the curve. BPM = heart rate beats per minute. SF-DCM = number of spontaneous fluctuations of skin conductance calculated with the dynamic causal modelling algorithm. HF = heart rate variability (high frequency). SF-MP = number of spontaneous fluctuations of skin conductance calculated with the matched pursuit algorithm. RMSSD = heart rate variability (root mean square of successive differences between normal heartbeats). RP = respiration period. SCL = skin conductance level. Outliers in physiological response indices were identified with the first (Q1) and third quartile (Q3) and the interquartile range (IQR = Q3-Q1). Values above Q3+ 1.5xIQR or below Q1 - 1.5xIQR were considered as outliers and excluded from the physiological data analyses. | | | | | |

| **Table S3. Post-hoc comparisons of physiological responses to intrusion provocation task between doxycylcine and placebo** | | | | | | | | | | | | | | | |
| --- | --- | --- | --- | --- | --- | --- | --- | --- | --- | --- | --- | --- | --- | --- | --- |
| Comparisons | RP |  |  |  |  | AUC |  |  |  |  | DCM |  |  |  |  |
| *contrast* | *estimate* | *SE* | *df* | *t.ratio* | *p.value* | *estimate* | *SE* | *df* | *t.ratio* | *p.value* | *estimate* | *SE* | *df* | *t.ratio* | *p.value* |
| DOXYCYCLINE b1 - PLACEBO b1 | 0.12 | 0.24 | 88.68 | 0.51 | 1.000 | 0.03 | 0.08 | 111.92 | 0.33 | 1.000 | - 0.01 | 0.03 | 136.43 | - 0.19 | 1.000 |
| DOXYCYCLINE pics - PLACEBO pics | 0.28 | 0.24 | 88.68 | 1.16 | 1.000 | 0.23 | 0.08 | 111.92 | 2.86 | 0.102 | 0.11 | 0.03 | 136.43 | 3.18 | 0.022 |
| DOXYCYCLINE intrusion provocation task - PLACEBO ipt | 0.30 | 0.24 | 88.68 | 1.23 | 1.000 | 0.14 | 0.08 | 111.92 | 1.72 | 1.000 | 0.16 | 0.03 | 136.43 | 4.65 | 0.000 |
| DOXYCYCLINE b2 - PLACEBO b2 | 0.22 | 0.24 | 88.68 | 0.91 | 1.000 | 0.13 | 0.08 | 111.92 | 1.59 | 1.000 | 0.11 | 0.03 | 136.43 | 3.30 | 0.016 |
|  |  |  |  |  |  |  |  |  |  |  |  |  |  |  |  |
| DOXYCYCLINE b1 - DOXYCYCLINE pics | 0.37 | 0.13 | 174.00 | 2.96 | 0.088 | - 0.05 | 0.06 | 147.00 | - 0.81 | 1.000 | 0.06 | 0.03 | 123.00 | 2.17 | 0.318 |
| DOXYCYCLINE pics - DOXYCYCLINE ipt | 0.51 | 0.13 | 174.00 | 4.06 | 0.002 | - 0.08 | 0.06 | 147.00 | - 1.36 | 1.000 | - 0.09 | 0.03 | 123.00 | - 2.97 | 0.040 |
| DOXYCYCLINE intrusion provocation task - DOXYCYCLINE b2 | - 0.22 | 0.13 | 174.00 | - 1.75 | 1.000 | - 0.12 | 0.06 | 147.00 | - 2.08 | 0.631 | - 0.17 | 0.03 | 123.00 | - 5.94 | 0.000 |
| DOXYCYCLINE b1 - DOXYCYCLINE ipt | - 0.14 | 0.13 | 174.00 | - 1.10 | 1.000 | 0.03 | 0.06 | 147.00 | 0.55 | 1.000 | 0.15 | 0.03 | 123.00 | 5.14 | 0.000 |
| DOXYCYCLINE b1 - DOXYCYCLINE b2 | 0.08 | 0.13 | 174.00 | 0.64 | 1.000 | 0.15 | 0.06 | 147.00 | 2.63 | 0.180 | 0.32 | 0.03 | 123.00 | 11.08 | 0.000 |
| DOXYCYCLINE pics - DOXYCYCLINE b2 | 0.29 | 0.13 | 174.00 | 2.31 | 0.479 | - 0.19 | 0.06 | 147.00 | - 3.44 | 0.018 | - 0.26 | 0.03 | 123.00 | - 8.91 | 0.000 |
|  |  |  |  |  |  |  |  |  |  |  |  |  |  |  |  |
| PLACEBO b1 - PLACEBO pics | 0.22 | 0.12 | 174.00 | 1.78 | 1.000 | - 0.25 | 0.06 | 147.00 | - 4.44 | 0.000 | - 0.05 | 0.03 | 123.00 | - 1.74 | 0.653 |
| PLACEBO pics - PLACEBO ipt | 0.53 | 0.12 | 174.00 | 4.35 | 0.001 | - 0.17 | 0.06 | 147.00 | - 3.01 | 0.064 | - 0.04 | 0.03 | 123.00 | - 1.21 | 0.931 |
| PLACEBO intrusion provocation task - PLACEBO b2 | - 0.30 | 0.12 | 174.00 | - 2.45 | 0.352 | - 0.13 | 0.06 | 147.00 | - 2.31 | 0.405 | - 0.22 | 0.03 | 123.00 | - 7.36 | 0.000 |
| PLACEBO b1 - PLACEBO ipt | - 0.32 | 0.12 | 174.00 | - 2.57 | 0.261 | - 0.08 | 0.06 | 147.00 | - 1.42 | 1.000 | - 0.02 | 0.03 | 123.00 | - 0.53 | 1.000 |
| PLACEBO b1 - PLACEBO b2 | - 0.02 | 0.12 | 174.00 | - 0.12 | 1.000 | 0.05 | 0.06 | 147.00 | 0.88 | 1.000 | 0.20 | 0.03 | 123.00 | 6.82 | 0.000 |
| PLACEBO pics - PLACEBO b2 | 0.23 | 0.12 | 174.00 | 1.90 | 1.000 | - 0.30 | 0.06 | 147.00 | - 5.32 | 0.000 | - 0.26 | 0.03 | 123.00 | - 8.56 | 0.000 |
|  |  |  |  |  |  |  |  |  |  |  |  |  |  |  |  |
|  | **HF** |  |  |  |  | **SCL** |  |  |  |  | **MP** |  |  |  |  |
| ***contrast*** | ***estimate*** | ***SE*** | ***df*** | ***t.ratio*** | ***p.value*** | ***estimate*** | ***SE*** | ***df*** | ***t.ratio*** | ***p.value*** | ***estimate*** | ***SE*** | ***df*** | ***t.ratio*** | ***p.value*** |
| DOXYCYCLINE b1 - PLACEBO b1 | - 0.12 | 0.11 | 138.23 | - 1.06 | 1.000 | - 0.44 | 0.74 | 63.92 | - 0.60 | 1 | 0.00 | 0.03 | 113.50 | 0.14 | 1.000 |
| DOXYCYCLINE pics - PLACEBO pics | - 0.11 | 0.11 | 138.23 | - 0.95 | 1.000 | - 0.23 | 0.74 | 63.92 | - 0.32 | 1 | 0.07 | 0.03 | 113.50 | 2.30 | 0.348 |
| DOXYCYCLINE intrusion provocation task - PLACEBO ipt | - 0.04 | 0.11 | 138.23 | - 0.31 | 1.000 | 0.14 | 0.74 | 63.92 | 0.19 | 1 | 0.06 | 0.03 | 113.50 | 2.04 | 0.570 |
| DOXYCYCLINE b2 - PLACEBO b2 | - 0.05 | 0.11 | 138.23 | - 0.48 | 1.000 | 0.53 | 0.74 | 63.92 | 0.72 | 1 | 0.07 | 0.03 | 112.04 | 2.36 | 0.321 |
|  |  |  |  |  |  |  |  |  |  |  |  |  |  |  |  |
| DOXYCYCLINE b1 - DOXYCYCLINE pics | - 0.10 | 0.08 | 180.00 | - 1.31 | 1.000 | - 0.11 | 0.27 | 156.00 | - 0.41 | 1 | 0.03 | 0.02 | 135.17 | 1.33 | 1.000 |
| DOXYCYCLINE pics - DOXYCYCLINE ipt | - 0.49 | 0.08 | 180.00 | - 6.18 | 0.000 | 0.35 | 0.27 | 156.00 | 1.29 | 1 | - 0.02 | 0.02 | 135.17 | - 0.91 | 1.000 |
| DOXYCYCLINE intrusion provocation task - DOXYCYCLINE b2 | 0.37 | 0.08 | 180.00 | 4.63 | 0.000 | - 0.38 | 0.27 | 156.00 | - 1.38 | 1 | - 0.13 | 0.02 | 135.17 | - 5.76 | 0.000 |
| DOXYCYCLINE b1 - DOXYCYCLINE ipt | 0.39 | 0.08 | 180.00 | 4.87 | 0.000 | - 0.46 | 0.27 | 156.00 | - 1.70 | 1 | 0.05 | 0.02 | 135.17 | 2.24 | 0.375 |
| DOXYCYCLINE b1 - DOXYCYCLINE b2 | 0.02 | 0.08 | 180.00 | 0.24 | 1.000 | - 0.09 | 0.27 | 156.00 | - 0.32 | 1 | 0.18 | 0.02 | 135.17 | 8.00 | 0.000 |
| DOXYCYCLINE pics - DOXYCYCLINE b2 | - 0.12 | 0.08 | 180.00 | - 1.56 | 1.000 | - 0.02 | 0.27 | 156.00 | - 0.09 | 1 | - 0.15 | 0.02 | 135.17 | - 6.67 | 0.000 |
|  |  |  |  |  |  |  |  |  |  |  |  |  |  |  |  |
| PLACEBO b1 - PLACEBO pics | - 0.12 | 0.08 | 180.00 | - 1.47 | 1.000 | - 0.32 | 0.26 | 156.00 | - 1.22 | 1 | - 0.03 | 0.02 | 135.17 | - 1.60 | 1.000 |
| PLACEBO pics - PLACEBO ipt | - 0.42 | 0.08 | 180.00 | - 5.28 | 0.000 | 0.73 | 0.26 | 156.00 | 2.76 | 0.161 | - 0.03 | 0.02 | 135.17 | - 1.29 | 1.000 |
| PLACEBO intrusion provocation task - PLACEBO b2 | 0.35 | 0.08 | 180.00 | 4.39 | 0.000 | 0.01 | 0.26 | 156.00 | 0.05 | 1 | - 0.12 | 0.02 | 136.94 | - 5.50 | 0.000 |
| PLACEBO b1 - PLACEBO ipt | 0.31 | 0.08 | 180.00 | 3.81 | 0.004 | - 1.05 | 0.26 | 156.00 | - 3.98 | 0.003 | - 0.01 | 0.02 | 135.17 | - 0.31 | 1.000 |
| PLACEBO b1 - PLACEBO b2 | - 0.05 | 0.08 | 180.00 | - 0.57 | 1.000 | - 1.06 | 0.26 | 156.00 | - 4.04 | 0.002 | 0.11 | 0.02 | 136.94 | 5.18 | 0.000 |
| PLACEBO pics - PLACEBO b2 | - 0.07 | 0.08 | 180.00 | - 0.89 | 1.000 | 0.74 | 0.26 | 156.00 | 2.82 | 0.143 | - 0.15 | 0.02 | 136.94 | - 6.79 | 0.000 |

*Note*. Table contains post-hoc comparisons of significant main effects of physiological responses in the INTRUSION PROVOCATION TASK tested with two-sided t-tests. Results are Holm-Bonferroni corrected. SE = Standard error. Df = degrees of freedom.

AUC = skin conductance area under the curve. BPM = heart rate beats per minute. DCM = number of spontaneous fluctuations of skin conductance calculated with the dynamic causal modelling algorithm. HF = heart rate variability (high frequency). MP = number of spontaneous fluctuations of skin conductance calculated with the matched pursuit algorithm. RMSSD = heart rate variability (root mean square of successive differences between normal heartbeats). RP = respiration period. SCL = skin conductance level. b1 = 5-min fixation cross immediately before task. pics = 22-sec of reminder images in chronological order. intrusion provocation task = 2-min-phase of intrusion monitoring with eyes closed, b2 = 5-min fixation cross immediately after task. Outliers in physiological response indices were identified with the first (Q1) and third quartile (Q3) and the interquartile range (IQR = Q3-Q1). Values above Q3+ 1.5xIQR or below Q1 - 1.5xIQR were considered as outliers and excluded from the physiological data an

*Fixed Latency Skin Conductance Response*

As the IPT phase contained 11 reminder pictures, each presented for 2 seconds, the picture-evoked SCR might suppress SF (5). Hence, we also modelled in additional and exploratory analyses fixed latency SCR evoked by each picture onset and compared the estimates to those evoked at non-stimulus onsets in the rest condition during a time period of the same length. This revealed that the first pictures elicited an increased skin conductance response, but not later pictures, which could have led to undetectable skin conductance fluctuations.

Figure S1


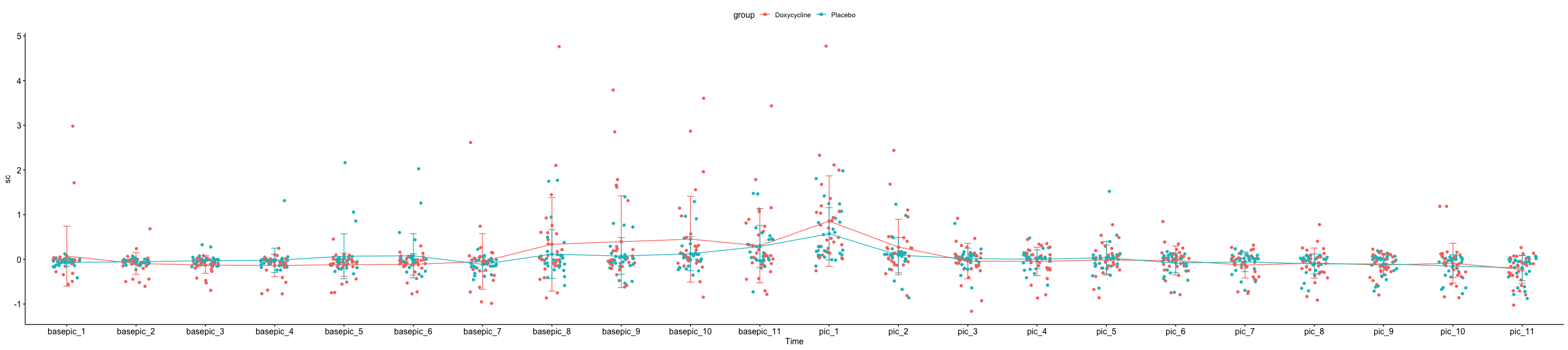


Note. Fixed latency skin conductance response during intrusion provocation task.

## Appendix

**References**

1. Ojala KE, Bach DR. Measuring learning in human classical threat conditioning: Translational, cognitive and methodological considerations. Neurosci Biobehav Rev. Juli 2020;114:96–112.

2. Bach DR, Friston KJ. Model‐based analysis of skin conductance responses: Towards causal models in psychophysiology. Psychophysiology. Januar 2013;50(1):15–22.

3. Luke SG. Evaluating significance in linear mixed-effects models in R. Behav Res Methods. August 2017;49(4):1494–502.

4. Schielzeth H, Dingemanse NJ, Nakagawa S, Westneat DF, Allegue H, Teplitsky C, u. a. Robustness of linear mixed‐effects models to violations of distributional assumptions. Sutherland C, Herausgeber. Methods Ecol Evol. September 2020;11(9):1141–52.

5. Gerster S, Namer B, Elam M, Bach DR. Testing a linear time invariant model for skin conductance responses by intraneural recording and stimulation. Psychophysiology. Februar 2018;55(2):e12986.

**Figures**

**Fig. A Distribution of Model Residuals Linear mixed-effects model High Frequency TFP**

**
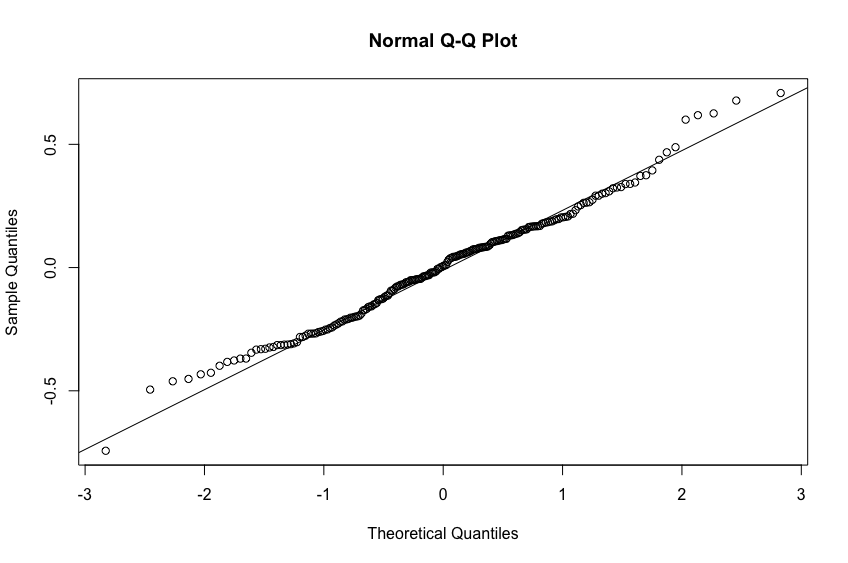
**

*Note.* The distribution of the residuals used to calculate group*time effects on high frequency during at the assessment at the trauma film paradigm are normally distributed.

**Fig. B Intrusion Provocation Task High Frequency Distribution of Residuals**

**
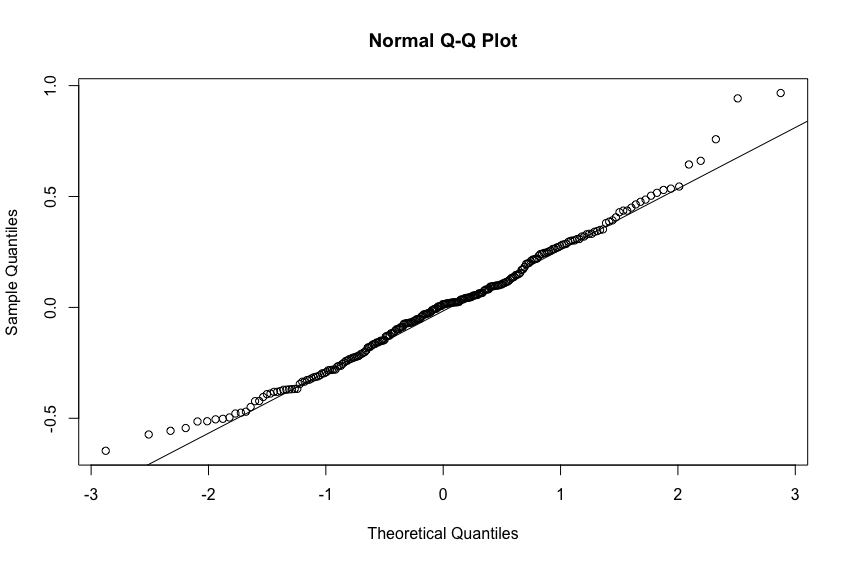
**

*Note.* The distribution of the residuals used to calculate group*time effects on high frequency during the assessment at the intrusion provocation task are normally distributed.
